# EarSet: A Multi-Modal Dataset for Studying the Impact of Head and Facial Movements on In-Ear PPG Signals

**DOI:** 10.1038/s41597-023-02762-3

**Published:** 2023-12-01

**Authors:** Alessandro Montanari, Andrea Ferlini, Ananta Narayanan Balaji, Cecilia Mascolo, Fahim Kawsar

**Affiliations:** 1 Nokia Bell Labs, Cambridge, UK; 2 University of Cambridge, Cambridge, UK; 3 National University of Singapore, Singapore, Singapore

**Keywords:** Scientific data, Electrical and electronic engineering, Computer science

## Abstract

Photoplethysmography (PPG) is a simple, yet powerful technique to study blood volume changes by measuring light intensity variations. However, PPG is severely affected by motion artifacts, which hinder its trustworthiness. This problem is pressing in earables since head movements and facial expressions cause skin and tissue displacements around and inside the ear. Understanding such artifacts is fundamental to the success of earables for accurate cardiovascular health monitoring. However, the lack of in-ear PPG datasets prevents the research community from tackling this challenge. In this work, we report on the design of an ear tip featuring a 3-channels PPG and a co-located 6-axis motion sensor. This, enables sensing PPG data at multiple wavelengths and the corresponding motion signature from both ears. Leveraging our device, we collected a multi-modal dataset from 30 participants while performing 16 natural motions, including both head/face and full body movements. This unique dataset will greatly support research towards making in-ear vital signs sensing more accurate and robust, thus unlocking the full potential of the next-generation PPG-equipped earables.

## Background & Summary

Monitoring vital signs, such as cardiovascular functions, heart rate, oxygen saturation, and blood pressure, through Photoplethysmography (PPG) is common across wearables like smartwatches^1^. Photoplethysmography, as suggested by its name, is an optical technique used to infer blood volumetric changes in the peripheral circulation. PPG is indeed a remarkable signal, which not only carries a wealth of clinical information (such as heart rate, heart rate variability, blood oxygen saturation, respiration rate, blood pressure, and artery characteristics^2–4^), but can also be used for non-medical applications such as authentication^5^ and drowsiness detection^6^.

At the same time, the past years have witnessed the widespread diffusion of a new family of wearables: smart earbuds (also known as *earables*). Earables are mostly known for their leisure applications (e.g., Apple AirPods), showing their capability in enhancing the user’s auditory experience with, for instance, noise cancellation and spatially aware audio. However, they are also gaining traction, across the research community, for personal health monitoring^7–10^, activity recognition^11–14^, authentication^15^, and navigation^16^. Earables are posed to revolutionize the mobile health (*mHealth*) market^17^. Thanks to their proximity to the human sensorium (i.e., brain, ears, eyes, mouth, and nose), earables are in a unique position with respect to other, more traditional, wearables like smartwatches^18^. Indeed, earables have allowed the research community to investigate a number of novel applications such as monitoring cerebral activity during sleep through electroencephalography (EEG)^19^, eye-movements^20^ and tracking eating episodes, dietary and swallowing activities^21^.

Notably, previous works have explored PPG sensing in or around the ear focusing on specific applications. However, PPG signal acquisition is particularly challenging in the presence of either ambient light or motion. While the former can be mitigated by ambient light rejection modules (often already implemented in hardware), there still is no unanimously agreed technique to mitigate the latter without a considerable loss of information. Earlier works considered only motion artifacts (MA) arising from body movements, like walking or running^22–25^. However, the head and facial region consist of an intricate mesh of muscles and blood vessels that contract and relax with each of their movements. This induces unwanted noise and motion artifacts in the PPG signals recorded from the ear. The interaction between these motions and the signals recorded from in-ear PPG sensors remains entirely unexplored.

Very few openly available datasets feature PPG data from the ear^26,27^. However, there are no publicly available datasets that explore the effect of facial expressions and head movements on earables. Table 1 presents an overview of existing datasets in the literature that provide PPG signals collected at various body locations. Recently^27^, proposed a solution for how motion artifacts can be removed for accurate heart rate and blood pressure estimation with PPG sensors placed on the ear lobes. However, they only study the effect of body motion artifacts on the acquired PPG signals. Hence, there is a strong need for an open-source dataset studying the effect of facial motions on in-ear PPG signals.

**Table 1:** PPG datasets publicly available for motion artifact studies.

Datasets	PPG sensor location	Motion being studied	Additional sensor data	Number of participants
Activity monitoring^44^	Wrist	Squat exercises, stepper exercises, and resting	3-channels PPG	7
			3-axis accelerometer	
PPG DaLiA^45^	Wrist	Daily life activities like sitting, walking, cycling, driving, working, etc.	3-channels PPG	15
			Electrocardiogram (ECG)	
			Electrodermal activity (EDA)	
			3-axis accelerometer	
			Respiration rate	
			Body temperature	
Effect of exercises on PPG signals^46^	Wrist	Walking, running, and biking	3-channels PPG	23
			Chest ECG	
			3-axis accelerometer	
			3-axis low noise accelerometer	
			3-axis gyroscope	
Motion artifact removal in PPG signals (IEEE signal processing cup)^47^	Wrist	Random physical exercises without labels	3-channels PPG	12 (training dataset) 10 (test dataset)
			Chest ECG	
			3-axis accelerometer	
Motion artifact cancellation^48^	Wrist	Walking and running	3-channels PPG	24
			Chest ECG	
			3-axis accelerometer	
			3-axis gyroscope	
WESAD (Stress detection)^49^	Wrist and Chest	Intense physical activity and mental exercises to induce stress	Wrist PPG	17
			Wrist accelerometer	
			Wrist electrodermal activity (EDA)	
			Body temperarture	
			Chest ECG	
			Chest accelerometer	
			Chest EMG	
			Chest Respiration	
BIDMC dataset^50^	Finger	No exercise involved	Finger PPG	53
			Pneumography (Respiration)	
FatigueSet^26^	In-Ear	Running on a treadmill to induce physical fatigue	In-Ear PPG	12
			In-Ear IMU sensor	
			Chest ECG	
			Chest Respiration sensor	
			Wrist PPG	
			Wrist EDA	
			Wrist IMU	
			Body temperature sensor	
Motion tolerant heart rate and Blood pressure monitoring^27^	Outside ear	Exercising on a bike	Ear PPG	14
			Ear ECG	
			Ambulatory blood pressure monitor	
EarSet	Stereo In-ear	16 different facial and head motions	In-Ear PPG (Both Left and right)	30
			In-Ear IMU (Both Left and right)	
			Chest band ECG	
			Chest band Respiration sensor	

To this end, this work aims at *providing the research community with a novel, multi-modal, dataset, which, for the first time, will allow studying of the impact of body and head/face movements on both the morphology of the PPG wave captured at the ear, as well as on the vital signs estimation*. To accurately collect in-ear PPG data, coupled with a 6 degrees-of-freedom (DoF) motion signature, we prototyped and built a flexible research platform for in-the-ear data collection. The platform is centered around a novel ear-tip design which includes a 3-channels PPG (green, red, infrared) and a 6-axis (accelerometer, gyroscope) motion sensor (IMU) co-located on the same ear-tip. This allows the simultaneous collection of spatially distant (i.e., one tip in the left and one in the right ear) PPG data at multiple wavelengths and the corresponding motion signature, for a total of 18 data streams. Inspired by the Facial Action Coding Systems (FACS)^28^, we consider a set of potential sources of motion artifact (MA) caused by natural facial and head movements. Specifically, we gather data on 16 different head and facial motions, including head movements (nodding, shaking, tilting), eyes movements (vertical eyes movements, horizontal eyes movements, brow raiser, brow lowerer, right eye wink, left eye wink), and mouth movements (lip puller, chin raiser, mouth stretch, speaking, chewing). We also collect motion and PPG data under activities of different intensities, which entail the movement of the entire body (walking and running). Together with in-ear PPG and IMU data, we collect several other vital signs such as heart rate, heart rate variability, and breathing rate from a medical-grade chest device.

With approximately 17 hours of data from 30 participants of mixed gender and ethnicity (mean age: 28.7 years, standard deviation: 5.3 years), our dataset empowers the research community to analyze the morphological characteristics of in-ear PPG signals with respect to **motion,** **device positioning** (left ear, right ear), as well as a set of **configuration parameters** and their corresponding data quality/power consumption trade-off. We envision such a dataset could open the door to innovative filtering techniques to mitigate, and eventually eliminate, the impact of MA on in-ear PPG. We ran a set of preliminary analyses on the data and observe statistically significant morphological differences in the PPG signal across different types of motions when compared to a situation where there is no motion. These preliminary results represent the first step towards the detection of corrupted PPG segments and show the importance of studying how head/face movements impact PPG signals in the ear.

To the best of our knowledge, this is the first in-ear PPG dataset that covers a wide range of full-body and head/facial motion artifacts. Being able to study the signal quality and motion artifacts under such circumstances will serve as a reference for future research in the field, acting as a stepping stone to fully enable PPG-equipped earables.

## Methods

To accurately analyze the in-ear PPG motion artifacts arising from head and facial motions, we design a controlled experiment and ask participants to perform a set of pre-defined body, head, and facial motions. We opted for a controlled study since it enables running a detailed analysis of the phenomenon under investigation and it is suitable for the reproducibility of the data collection procedure. In this section, we provide details regarding the study population, data collection procedure, and the collected data.

### Participants

Thirty individuals (18 males, 12 females, 20–49 years of age, mean age: 28.7 years, standard deviation: 5.3 years) were recruited and voluntarily took part in the study. None of the participants had any underlying heart or respiratory condition and were in good health at the time of the study. We used the standard Fitzpatrick skin tone scale^29^ to group our participants based on skin tone. The scale includes 6 types, 1 being the lightest and 6 being the darkest. Despite being dominated by type 2 skin tone (n = 18), our dataset includes type 1 (n = 2), type 3 (n = 4), type 4 (n = 4), and type 5 (n = 2) skin tone groups.

Before taking part in the study, the investigators briefed all the participants who then gave their written consent (by completing an informed consent form) to release their data publicly. Every participant received a gift card as compensation upon completion of the study. The study was approved by the ethics board of the department of Computer Science and Technology at the University of Cambridge (application number 1873).

### Devices and setup

**Fig. 1:**
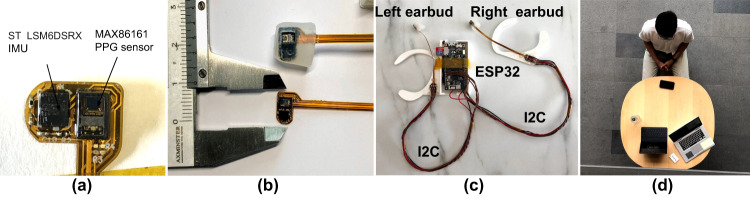
(**a**) Flexible PCB implementation of our earbud featuring MAX86161 PPG sensor and a co-located ST LSM6DSRX IMU. (**b**) An in-ear soft earbud was realized by embedding the in-ear flexible PCB board into a transparent silicone mold. (**c**) Head-worn data acquisition device consisting of an ESP32 microcontroller collecting data from in-ear PPG and IMU sensors in the left and right ear. (**d**) A participant wearing our earbud-based prototype and taking part in the data collection protocol.

**Fig. 2:**
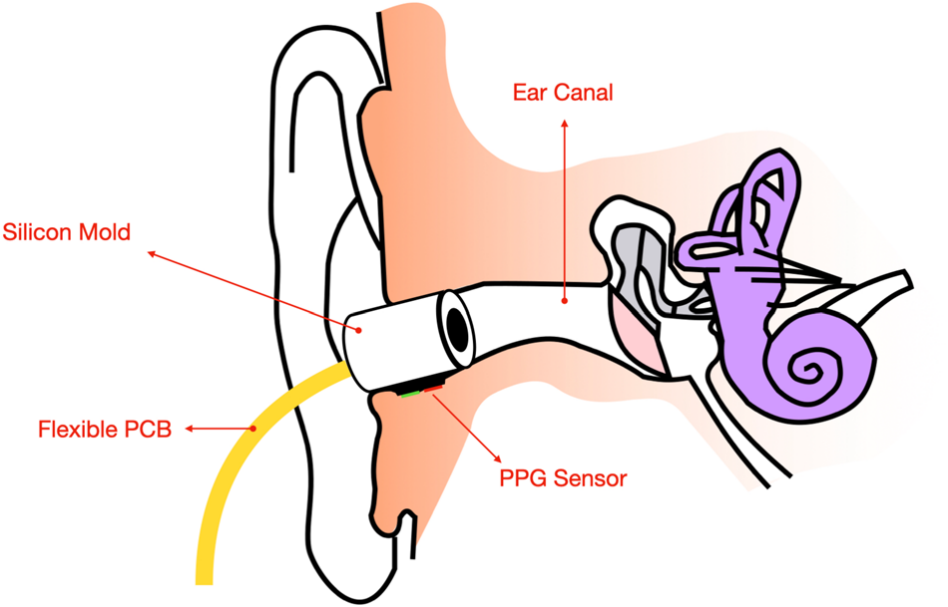
Representation of the custom-built PPG ear tip inside the ear canal.

Given the lack of existing open-source in-ear PPG platforms, we designed a custom head-worn prototype (see Fig. 1c) to collect in-ear PPG signals with established and affordable hardware components. The prototype consists of an ESP32 microcontroller collecting sensor data from both the left and right ears. In order to facilitate the PPG signal acquisition from inside the ear (Fig. 2), we fabricated a flexible PCB board consisting of a MAXM86161 (https://www.maximintegrated.com/en/products/sensors/MAXM86161.html) PPG sensor and ST-LSM6DSRX (https://www.st.com/en/mems-and-sensors/lsm6dsrx.html) IMU as shown in Fig. 1a. The flexible PCB board is interfaced via the I2C protocol to the ESP32 microcontroller for data acquisition. MAXM86161 is a well-known 3-channels PPG sensor (green - 520 to 550 nm, red - 660 nm, infrared - 880 nm) catered for in-ear sensing applications. The IMU continuously records 3-axis accelerometer and 3-axis gyroscope data to provide motion signals for in-ear motions occurring while making facial expressions or head movements. Both sensors are sampled at a frequency of 100 Hz. As shown in Fig. 1b, the flexible PCB containing the PPG sensor and the IMU was coated with soft silicone to resemble a typical ear tip to provide comfort while wearing the device, as well as remain firm within the ear during various face/head motions. We used a transparent soft silicone gel to prevent any distortions in the acquired PPG signals. Figure 2 reports a drawing of the device when placed inside the ear canal.

PPG signal quality is not only affected by motion but also by the sensor’s configuration. Typically, sensors allow changing several parameters which affect the acquired signal and consequently the power consumed by the sensor. Given this trade-off, often, optimal parameters for signal quality are not the most efficient in terms of power consumption. To explore this aspect of PPG sensing, we configured our device to change the sensor parameters every 30 seconds. This way, by collecting data for 2 minutes for each motion session we could cycle through 4 different sets of configurations (Table 2). In particular, the MAXM86161 allows changing of three parameters: *LED current* which determines the brightness of the three LEDs, *pulse width* which is the time each LED is kept on during measurement, and *the integration time* which is the period during which the photodiode is active and sampling the reflected light. Notice that pulse width and integration time cannot be controlled individually and only 4 combinations of the two parameters are available in the sensor. As shown in Table 2, we have chosen 4 configurations that offer distinct power consumption profiles and should result in diverse SNR characteristics.

**Table 2:** PPG parameters and relative sensor current draw.

Conf.	LED Current (mA)	Pulse Width (us)	Integration Time (us)	Current Draw (mA)
1	16	21.3	14.8	1.62
2	32	21.3	14.8	1.81
3	16	123.8	117.3	2.66
4	32	123.8	117.3	3.78

On the other hand, as a ground truth to collect vital signs from a reliable source, not affected by motion artifacts, we rely on a Zephyr Bioharness 3.0 (https://www.zephyranywhere.com/), a portable, medical-grade (FDA approved^30^), ECG chest band. The participants wore the portable ground truth ECG band on their chests for the whole experiment.

### Data collection protocol

**Fig. 3:**
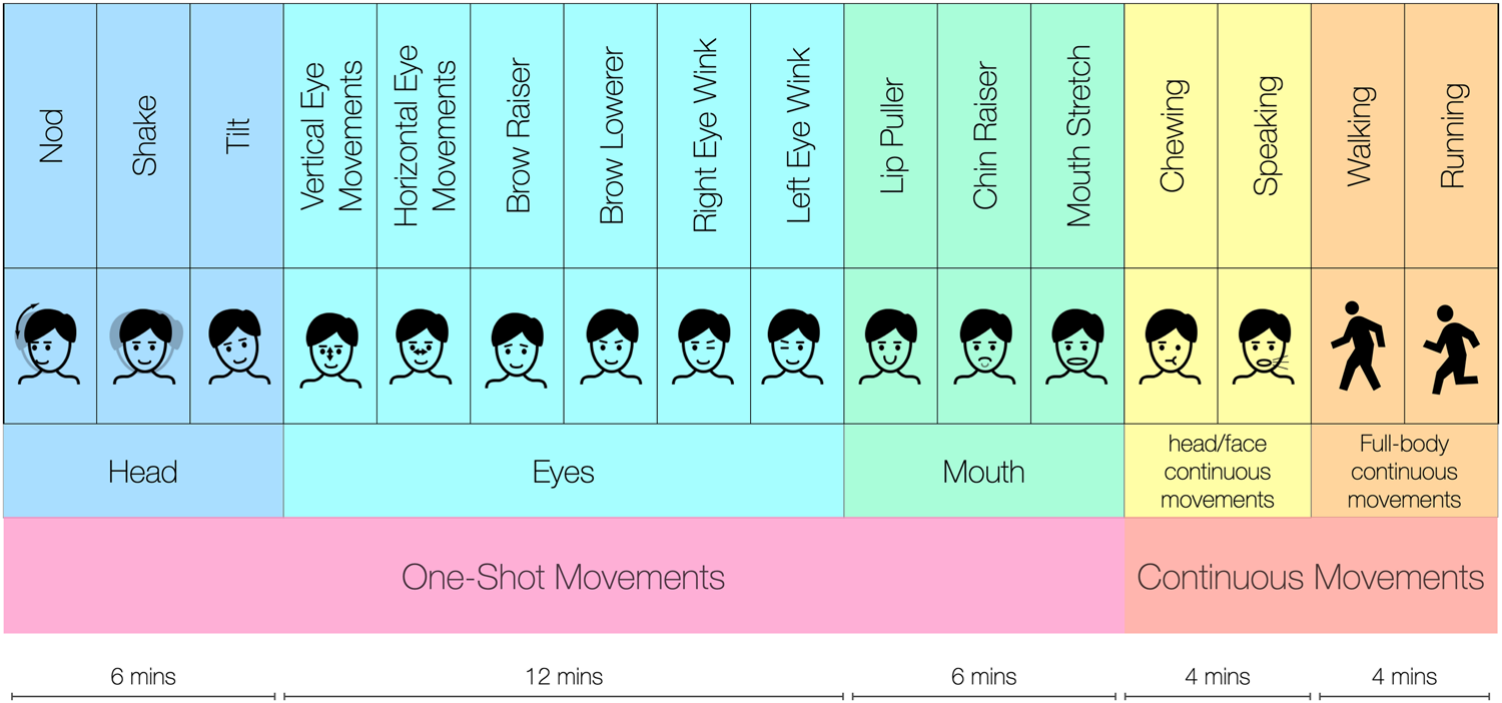
Summary of the data collection protocol following a 2 minutes long still baseline. Each activity was performed for 2 minutes.

After being briefed about the study, the participants wore our in-ear data collection device on the head placing the ear-tips in the left and right ear canal (Fig. 1) and the Zephyr Bioharness 3.0 ECG chest band. As in several prior works^30,31^, the Zephyr acts as ground truth device in our data collection. Starting from a resting pose (participants sitting still without any motion), we progressively asked the participants to repetitively carry out individual movements. Notably, for the entire duration of each data collection session, one of the investigators stayed in the room with the participant (carefully observing social distancing and other COVID-19 precautions). We consider two main classes of motions: head/face movements and full-body movements. A summary of the data collection protocol (following the 2-minutes-long still baseline) is reported in Fig. 3. By looking at the inherent nature of the motions, head/face movements can be further categorized into *one-shot* and *continuous* movements.

1. **One-shot motions:** One-shot motions are not normally performed continuously, and they are often performed in normal social interactions as well as in the form of psychosomatic tics. The selection process for the one-shot motion artifacts was informed by both anatomy principles^28^ and previous work^32–34^. In building our dataset, we look at Action Units (AUs) that entail the movement of the head, the eyes (and the adjacent muscles), and the mouth. Specifically, we selected: *(1) nod; (2) shake; and (3) tilt* as **head** movements. The **eye** movements chosen were: *(4) vertical eye movements; (5) horizontal eye movements; (6) brow raiser; (7) brow lowerer; (8) right eye wink; and (9) left eye wink*. Finally, we investigated: *(10) lip puller; (11) chin raiser; and (12) mouth stretch* as **mouth** movements. We instructed the participants to repeat the one-shot movements roughly every 5 seconds.
2. **Continuous motions:** Besides, we also accounted for **head/face continuous movements** caused by common activities such as (*13) chewing; and (14) speaking*. Together with the one-shot movements, Continuous movements are quite unique to ear-worn devices. In fact, when performing these, the complex mesh of facial muscles moves substantially and, therefore, these activities are likely to cause significant deformations of the tissues in and around the ear.

**Fig. 4:**
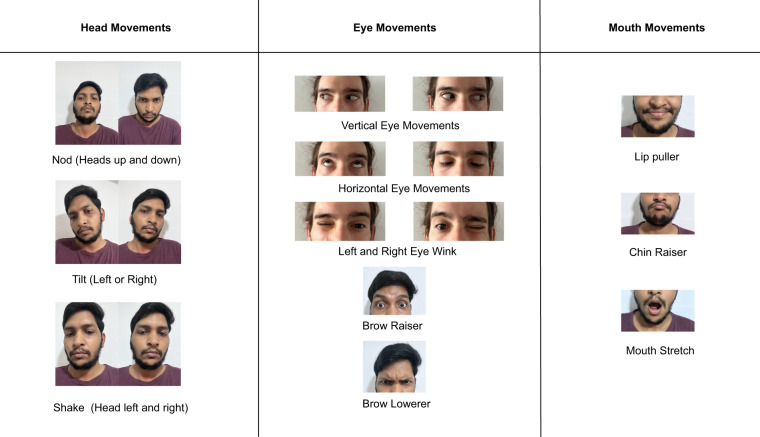
Summary of the Facial Action Units (subset of the FACS) considered in the dataset^65^. The individuals depicted provided consent for the open publication of the images.

Apart from head/face motions, we also considered **full-body activities** such as *(15) walking and (16) running*, which give rise to well-known sources of noise^35^ in the PPG signal. The list of all the considered motion artifacts is reported in Table 4 and pictured in Fig. 4. Notably, before performing each and every motion, the investigator demoed each and every gesture/activity to the participants. Ultimately, for all the conditions but the full-body movements (walking and running), we followed the wearable device validation guideline stipulated by the Consumer Technology Association^36^ and acquired PPG signals while seated in the upright position. During the *resting* condition, we instructed the participants to breathe normally without moving. The *speaking* condition consisted of a conversation with the investigator, where the participant described a recent event to the investigator. The *chewing* condition was assessed by recording PPG data while the participant was chewing gum. For the full-body motion conditions, the participants were asked to walk and run at a set pace on a treadmill. We set the treadmill’s speed at 5*kph* and 8*kph* while walking and running, respectively. For each motion condition, we recorded 2 minutes of data, automatically changing the configuration of the PPG parameters every 30 seconds using the values described earlier. The length of the sessions was carefully chosen to be long enough to yield good-quality vital signs and yet not too tedious/harmful to the participants.

### Collected data

We collected three types of data: (a) In-ear PPG signals from both left and right ear, (2) In-ear IMU signals from both left and right ear, and (3) Ground-truth heart rate data from Zephyr Bioharness 3.0 ECG chest band. Table 3 reports an overview of the characteristics of devices used to collect *EarSet* dataset. The table presents the type of data that was collected for each device as well as the sampling rate at which the data was collected. The table shows that *EarSet* contains data from 2 different devices (including an ECG ground truth as ground-truth information) placed on 2 unique body locations(in-ear and chest). The data from the accelerometer was available in both the body locations (ear and chest). Here now follows more details on the collected raw data.

1. **In-ear PPG signals**: The in-ear PPG signals (19-bit analog to digital converted PPG values from MAXM86161) were collected using our custom head worn prototype at a sampling frequency of 100 Hz. The timestamps (in milliseconds resolution) are available for each PPG signal sample from both the left and the right ear. For each motion artifact, the PPG signals were collected for 2 minutes. Every 30 seconds, the PPG configuration was changed in the order reported in Table 2. As explained earlier, PPG signals were collected at three different wavelengths–green (530 nm), red (660 nm), and infrared (880 nm).
2. **In-ear IMU signals**: The in-ear IMU signals (both 3-axis accelerometer and gyroscope) were collected simultaneously with PPG signals using our custom head worn prototype at a sampling frequency of 100 Hz. The timestamps (with a milliseconds resolution) are available for each IMU record from both the left and the right ear. The IMU signals were also recorded continuously for each motion artifact session.
3. **Zephyr ground-truth data**: The Zephyr Bioharness 3.0 was worn by the participants on the chest and used to collect the ground-truth data. Specifically, the Zephyr provides heart rate (bpm), heart rate variability (ms) and ECG R-R interval (ms) at a sampling frequency of 1 Hz. In addition, the Zephyr provides raw 3-axis accelerometer data collected at a sampling frequency of 100 Hz. We also collect posture information (in degrees) at a sampling frequency of 1 Hz. The chest band also has a breathing sensor from which raw breathing waveform (25 Hz) and breathing/respiration rate (1 Hz) were collected.

**Table 3:** Sensor data collected from each wearable device.

Sensor	Units/Range	Sampling Rate
**Earable prototype (one per ear)**		
Accelerometer	g {−2:+2}	100 Hz
Gyroscope	°/s {−500:+500}	100 Hz
PPG - green, infrared, and red channels	—	100 Hz
**Zephyr Bioharness 3.0 chest band**		
Heart Rate	beats per minute {25:240}	1 Hz
Breathing Rate	breaths per minute {3:70}	1 Hz
Core Temperature	degrees {10:60}	1 Hz
Posture	degrees from vertical {−180:180}	1 Hz
Activity vector magnitude units	g {−16:16}	1 Hz
Breathing Rate Amplitude	mV {0.25:15}	1 Hz
Heart Rate Variability	ms {0:65534}	1 Hz
ECG Amplitude	mV {0.25:15}	1 Hz

**Table 4:** List of the considered motion artifacts and corresponding action unit (AU).

Class	Muscle Group	One-Shot	Artifact Name	Action Units
Still	n/a	n/a	Still	n/a
Head/Face	Head	✓	Nod	AU 53, 54
		✓	Shake	AU 51, 52
		✓	Tilt	AU 55, 56
	Eyes	✓	Vertical Eyes Movements	AU 63, 64
		✓	Horizontal Eyes Movements	AU 61, 62
		✓	Brow Raiser	AU 1, 2
		✓	Brow Lowerer	AU 4
		✓	Right Eye Wink	AU 46
		✓	Left Eye Wink	AU 46
	Mouth	✓	Lip Puller	AU 12
		✓	Chin Raiser	AU 17
		✓	Mouth Stretch	AU 27
		✗	Chewing	AU 81
		✗	Speaking	AU 50
Full-Body	n/a	✗	Walking	n/a
		✗	Running	n/a

### PPG Features

Before delving into the detailed description of the dataset we collected, we summarize the signal processing techniques used with PPG signals. This lays the required signal processing foundation for understanding our dataset validation.

The most common biomarkers that can be derived from PPG are:

1. **Heart rate:** Peaks are detected from the AC component of the PPG signal to obtain the number of beats per minute. Typically the raw PPG signal is band-pass filtered between [0.4 Hz, 4 Hz] to obtain the AC component corresponding to the heart rate.
2. **Oxygen saturation (SpO**_**2**_**):** Oxygenated hemoglobin absorbs less red light whereas deoxygenated hemoglobin absorbs less infrared light. Thus, the ratio between red and infrared light intensities measured by the PPG sensor can be used to estimate SpO_2_ (R) as follows:
  1 $$R=\frac{{R}_{red}}{{R}_{infrared}}=\frac{A{C}_{red}/D{C}_{red}}{A{C}_{infarared}/D{C}_{infrared}}$$
3. **Heart rate variability (HRV)**: Heart rate variability is measured as the time difference between adjacent peaks in a PPG signal.
4. **Respiration rate (RR)**: A Synchrosqueezing transform (SST)^37^ is applied on the raw PPG signals to extract the respiration component (0.1–0.9 Hz). The number of peaks in the resulting respiration component of the PPG signals corresponds to the respiration rate (breaths per minute). Besides, there are other techniques^38^ using time domain and frequency domain features extracted from the PPG signal along with machine learning to estimate respiration rate.
5. **Blood pressure (BP)**: Blood pressure is typically computed by placing PPG sensors at two locations on the same artery (say, finger and wrist) and then measuring the time taken by the pulse wave to travel from one PPG location to the other (pulse transit time). BP is inversely proportional to the pulse transit time obtained by calculating the peak time shifts between the two PPG sensors. In recent years, many machine learning and deep learning techniques^39,40^ have also been proposed to estimate blood pressure from the extracted PPG signal features.

As seen from the above biomarkers, the time domain signal features from the PPG signal are essential to estimate heart rate, heart rate variability as well as blood pressure. Some of the frequency domain features help in differentiating a normal sinus rhythm from an arterial fibrillation (AF) signal or an abnormal heart signal. In addition to the above-mentioned features, many techniques use features extracted from the first-order derivatives and the second-order derivatives of the PPG signal to compute arterial stiffness^41^ and blood pressure^40^. The second-order derivative of a PPG signal provides useful information such as the location of the dicrotic notch, i.e., the time at which the diastolic peak occurs which provides information regarding the blood flow dynamics (systolic and diastolic phases).

**Fig. 5:**
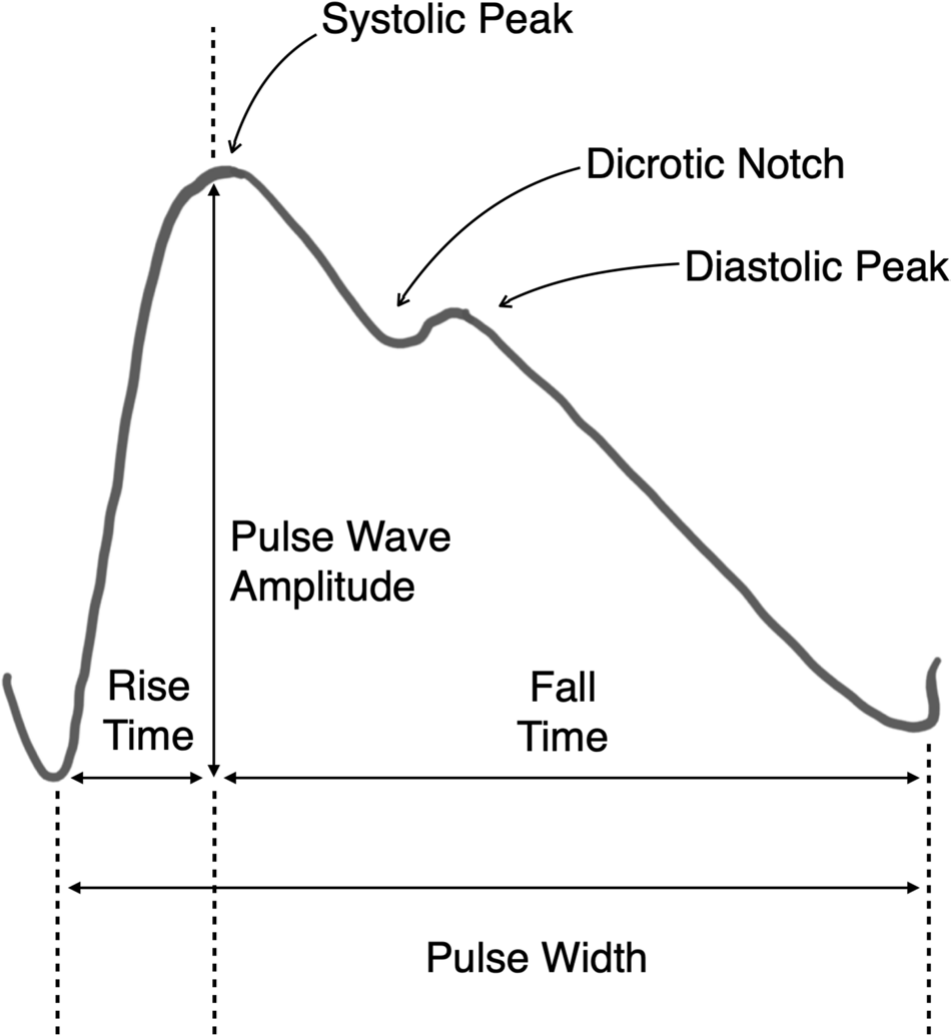
Typical time domain signal features extracted from a PPG signal.

Table 5 shows the main feature categories required for several critical health sensing applications. In addition to the PPG signal features mentioned earlier, useful physiological features marked in Fig. 5 can also be derived from the PPG signal^42^. The following list describes in more detail these main features which are also the ones we use in our technical validation of how various head and facial expressions affect in-ear PPG signals:

1. **Systolic phase:** The Amplitude of the systolic peak and the time at which the systolic peak is located in the PPG signal.
2. **Diastolic phase:** The Amplitude of the diastolic peak and the time at which the diastolic peak is located in the PPG signal.
3. **Ratio between systolic and diastolic phase:** It is an indicator of the abnormalities in blood pressure. It is also referred to as the Augmentation index or Reflection index.
4. **Pulse width:** It is the time between the beginning and end of a PPG pulse wave. It correlates with our heart’s systemic vascular resistance.
5. **Rise time:** The time between the foot of the PPG pulse and the systolic peak.
6. **Perfusion index (PI):** PI is the ratio of the pulsatile blood flow (AC component) to the non-pulsatile or static blood in peripheral tissue (DC component).
7. **Dominant frequency:** The dominant frequency of the PPG signal can be useful to give insights concerning the presence of artifacts at a different frequency outside the heart rate frequency band [0.4, 4 Hz].
8. **Spectral Kurtosis:** Also known as Frequency Domain Kurtosis, describes the distribution of the observed PPG signal frequencies around the mean and is a very useful indicator of the PPG signal quality.
9. **Peak-to-peak magnitude variance:** It is the variance of the difference between the pulse wave amplitude between two adjacent pulse waves.
10. **Peak-Time interval variance:** It is the variance of the pulse width between peaks of two adjacent PPG waves.

**Table 5:** Summary of PPG signal features essential for biomarkers as well as other health sensing applications.

Applications	AC Component	DC Component	Time domain signal features	Frequency domain signal features	First order derivative features	Second order derivative features
Vital sign sensing (HR^51^, SpO_2_^52^, BP^40^)	✓	✓	✓	✓	✓	✓
Heart rate variability (HRV)^53,54^	✓	✗	✓	✗	✗	✗
Respiration rate (RR)^55,56^	✓	✗	✗	✓	✗	✗
Sleep apnea^57,58^	✓	✗	✓	✗	✗	✗
Atrial Fibrillation^59,60^	✗	✗	✓	✓	✓	✓
Arterial Stiffness^41,61^	✗	✗	✓	✓	✓	✓
Energy expenditure^62^	✓	✗	✓	✓	✓	✓
Dehydration^63,64^	✗	✓	✗	✗	✗	✗

During the validation of our dataset, we show how the various motions and activities performed by the participants affect the features above. This demonstrates how head and facial motions could degrade the performance of health-related applications which rely on these features. We believe EarSet will help the research community in developing mitigation strategies for these motions and activities.

## Data Records

The raw data can be found at Zenodo^43^. Data of each participant has been anonymized with the alphanumeric format: P#. We refer to this as a participant identifier. The dataset contains a folder for each participant and an additional file, *Demographics.csv*, containing the demographics (e.g., gender, age) and skin tone of each participant in an anonymous format. Within each participant folder, there are two other folders, namely, *EARBUDS* and *ZEPHYR*, which contain the raw data obtained from each device during data collection. Table 6 provides an overview and description of the main files inside a participant folder.

**Table 6:** Description of the content of the folders named *P#* in the dataset. In this table, we explain only the most relevant files in the dataset.

Device	File	Column(s)	Description
**EARBUDS**	<ID>-<ACTIVITY>-imu-left.csv <ID>-<ACTIVITY>-imu-right.csv	timestamp	Timestamp in UNIX format with millisecond resolution.
		ax/gx	X-axis of accelerometer/gyroscope sensor.
		ay/gy	Y-axis of accelerometer/gyroscope sensor.
		az/gz	Z-axis of accelerometer/gyroscope sensor.
	<ID>-<ACTIVITY>-ppg-left.csv <ID>-<ACTIVITY>-ppg-right.csv	timestamp	Timestamp in UNIX format.
		green	PPG sensor green wavelength.
		ir	PPG sensor infrared wavelength.
		red	PPG sensor red wavelength.
**ZEPHYR**	<ID>_Summary.csv	Timestamp	Timestamp in UNIX format with millisecond resolution.
		HR	Heart rate measured from the ECG sensor.
		BR	Breathing rate is measured from a pressure sensor in the strap.
		CoreTemp	Core temperature.
		Posture	Posture: 0° = subject vertical, 90° = subject prone, −90° = subject supine, ±180° = subject inverted.
		Activity	Vector magnitude of the three axial acceleration magnitudes over the previous 1 second, sampled at 100 Hz.
		PeakAccel	Peak acceleration magnitude from the previous second.
		BRAmplitude	Breathing rate amplitude is used for internal development only.
		HRV	Heart rate variability.
		ECGAmplitude	Uncalibrated ECG amplitude measured from the peak of the R wave to the peak of the S wave of the QRS complex

<*ID>* represents the participant number from 0 to 29. <*ACTIVITY>* is the artifact name as listed in Table 4.

### Earbuds data

The IMU and PPG data are split into different files for each activity considered. The IMU sensor used the same configuration for the entire recording, while the PPG cycled through the four configurations described in Table 2. The transition before each configuration is marked by a line in the format #<timestamp>, current:<curr>, tint:<tint>, where <timestamp> is the UNIX time with milliseconds resolution, <curr> is the LED current in milli-Ampere and <tint> is the integration time in micro-seconds (this determines also the pulse width). All data points after this line have been collected with the new sensor configuration. Notice that the first configuration does not have such a line at the beginning.

To use the data collected from earbuds, one should first convert the raw ACC data to milli-g by multiplying it by 0.061 and the raw GYRO data to milli-dps (degrees per second) by multiplying with 17.5. This is to convert the raw data coming from the sensor from an integer format to a more usable format (i.e., milli-g and milli-dps). The PPG data does not require any conversion.

### Zephyr data

The data from the Zephyr Bioharness is directly pre-processed by the device and provided at a 1 Hz granularity. Hence, data from this device can be used as is. Notably, in some instances, the first and last few data-points recorded by the Zephyr might present some artifacts due to the user wearing/removing the device.

### Missing data

During the data collection, device malfunctions caused a minor loss of data. The PPG data relative to the mouth stretch activity for *P0* and *P27* is missing. Similarly, sensor configuration #4 is missing for *P9* for the nod activity. In addition, the BRAmplitude data field recorded by the Zephyr is not present for users *P17*, *P26*, *P27*, *P28*, and *P29*. Finally, users *P3*, *P4*, *P7*, *P8*, and *P10* have corrupted Zephyr data (notably, their IMU and PPG data from our prototype are still perfectly usable).

## Technical Validation

In this section, we perform a preliminary analysis of the collected data to evaluate its technical validity. We independently processed the PPG signals from the 3 channels (green, red, infrared) recorded from the left and the right ears. The acquired PPG signals from the left and right ear were aligned in the time axis and stored in Pandas Data Frames. Each Data Frame is then re-sampled at 100 Hz to ensure a consistent sampling rate. The start and the end of each Data Frame were trimmed to ensure that each data frame has the same length. Note that our preliminary exploration only focuses on the 4th set of LED configuration parameters (LED current 32*mA*; pulse width 123.8*μs*; integration time 117.3*μs*), as described in our Methods.

### Dataset outlook and template matching

**Fig. 6:**
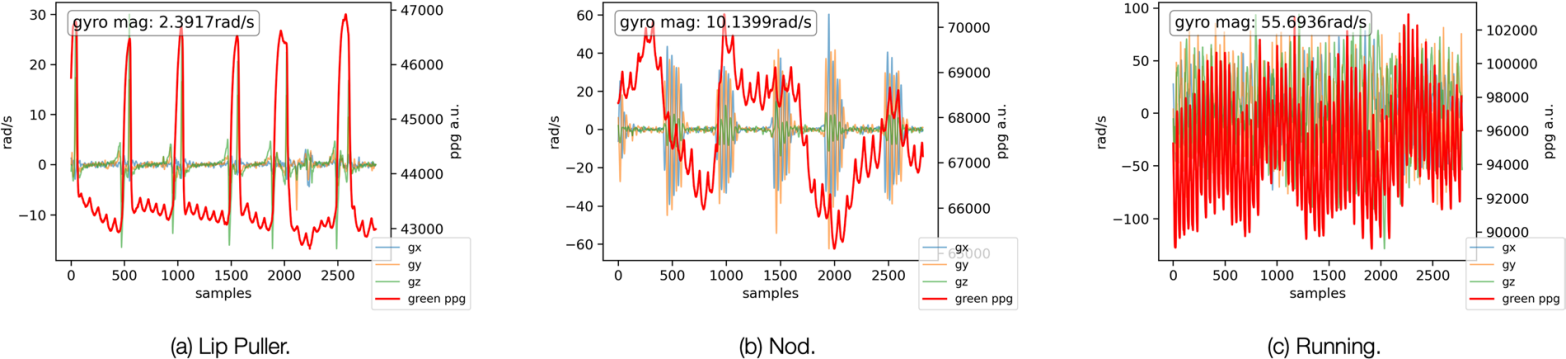
Samples of green PPG and IMU (gyroscope) data under different motion artifacts.

Firstly, we analysed EarSet to study how each facial motion artifacts appear unique in the collected in-ear PPG signals. In Fig. 6, we can appreciate at a glance how two diverse facial movements, such as lip puller (a) and nod (b), have a very different impact on the PPG trace when compared to a full-body movement like running (c)–in which the signal is dominated by the running cadence rather than by the cardiac signal. Notably, we can observe substantial differences even among the two facial movements: while the impact of the lip puller appears very localized and aligned with the motion (as we can see from the variations along the gyroscope axes), the nod seems to have a more prolonged impact on the DC component of PPG trace. By manually inspecting the data, we noticed that for a few [participant, motion] combinations, the PPG was not affected by artifacts. In particular, the vertical and horizontal movement of the eyes did not cause any artifact on the PPG signals. This is due to the limited involvement of the facial muscles, especially of those near the ears, during eye movements. Similarly, for the left and right eye wink motions, some participants could not perform the motion with both eyes or not at all. In other cases, the wink was subtle and hence did not result in any artifact in the corresponding PPG signal. For the rest of the analysis, we filtered out these [participant, motion] combinations for which the PPG was not affected by motion.

**Fig. 7:**
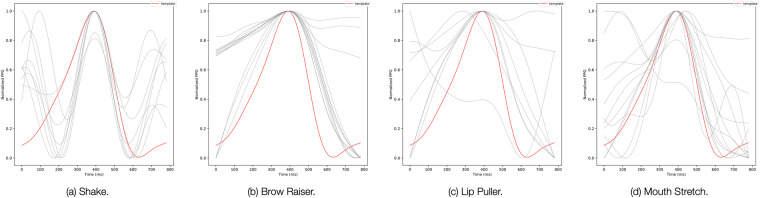
Template matching of PPG pulses from user 12 for four different motions. The red line represents the template pulse computed with data from the still condition. The gray lines are the pulses from different motion conditions.

To deepen our investigation, and gain a better visual understanding of how the various motion artifacts affect the morphology of the PPG pulses, we relied on a template matching analysis^42^. In doing so, we crafted a template pulse by taking the average of all the pulses of each user when still. We then plot the template pulse in red and use it as a reference against all the PPG pulses present in each motion session (plotted in gray). Figure 7 depicts the template matching analysis for shake (a), brow raiser (b), lip puller (c), and mouth stretch (d). The plots show how each of the considered movements affects the morphology of the PPG pulse differently, resulting in subtle, yet notable artifacts. Many applications rely on morphological features computed on the PPG signals^42^. Hence, such artifacts in the morphology of each pulse could lead to erroneous vitals estimation. We believe that our dataset represents a good resource for a more in-depth study and characterization of this issue for an emerging class of devices–earables equipped with heath-related sensors.

### Handcrafted metrics extraction from EarSet

We sought to proceed with our exploration of the dataset by extracting handcrafted features commonly derived from PPG signals for various health sensing applications listed in our Methods. For all the PPG signal metrics excluding Perfusion Index, we apply a 4th-order Butterworth band-pass filter (low-cut =0.4*Hz*, high-cut =4*Hz*) for signal smoothening. To facilitate a fair comparison of the PPG signal metrics for each facial motion artifact available in EarSet, we normalized their values using a standard min-max normalization. We chose to independently normalize the metric values for each user’s motions artifacts. Specifically, normalizing every user independently allows us to retain the subject-dependent motion artifact characteristics as well as the unique blood vessel morphology of each user.

**Fig. 8:**
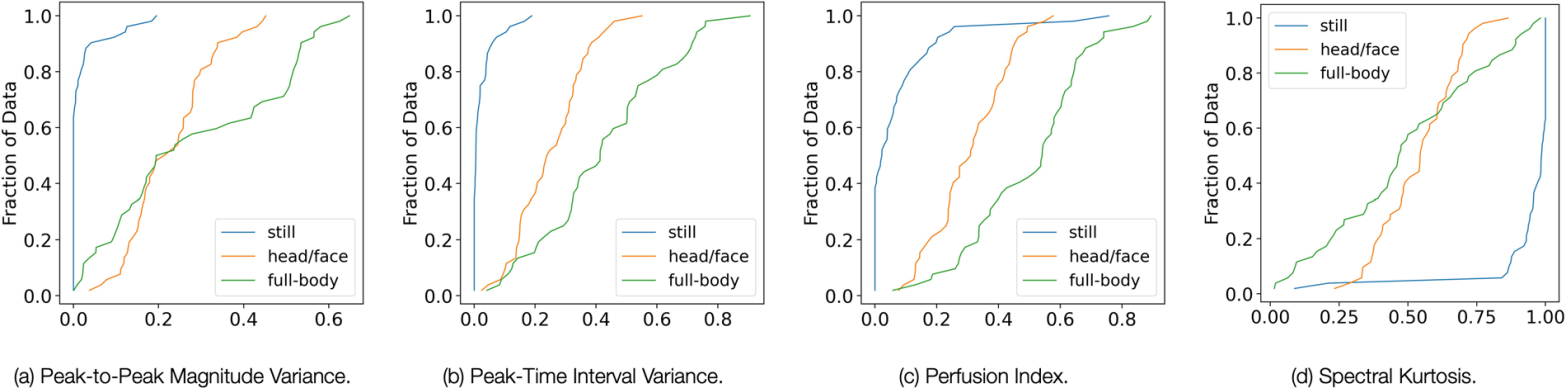
Empirical Cumulative Distribution Function (ECDF) of how the various classes of motion artifacts impact some of the handcrafted metrics extracted from PPG.

Figure 8 reports the empirical cumulative distribution function (ECDF) of how head/face and full-body movements impact the Peak-to-Peak Magnitude Variance (a), Peak-Time Interval Variance (b), Perfusion Index (c), and the Spectral Kurtosis (d) of the in-ear PPG signal. Similar patterns can be observed for other metrics. For this analysis, we considered the normalized PPG signal metrics computed from both the left and the right ear for all the users. We can observe that the PPG signal metrics for the “still” situation remain consistent across the entire population. On the other hand, the facial(head/face) and full-body movements appear to have more widespread distributions as well as different patterns. This is especially true for full-body movements. Notably, the findings of the spectral kurtosis analysis (d) are also aligned to the literature^42^, showing higher values for clean PPG signal. This can be explained by the presence of sharper peaks in the Fourier spectrum of clean (still in our case) PPG. These preliminary results suggest that different motion categories (i.e., head/face and full-body) create diverse artifacts in the PPG signal, and therefore it might be necessary to adopt dedicated approaches when applying signal filtering techniques. Our preliminary analysis of EarSet show that our dataset is a good source to start exploring this avenue.

**Fig. 9:**
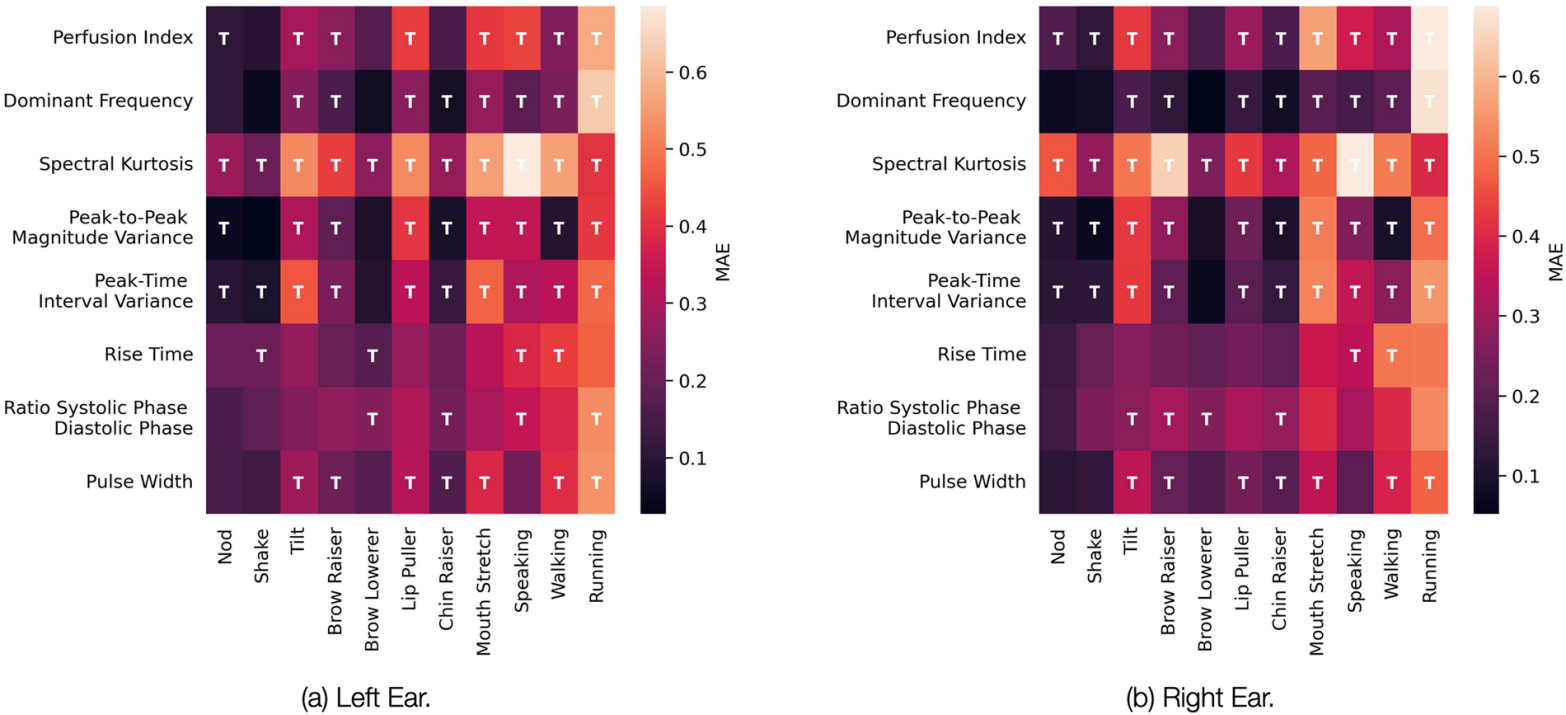
Heatmaps of how the various motion artifacts impact the handcrafted metrics extracted from the green PPG signal ((**a**) left ear; (**b**) right ear). The values reported in the heatmaps are the Mean Absolute Error (MAE) with respect to the still baseline. The heatmaps’ cells are annotated with a T whenever there is a statistically significant difference between the still baseline signal and the MA-corrupted one (*p* < 0.05).

Finally, We studied whether it is possible to spot differences between the individual motions using the collected PPG signals in EarSet. We began by looking at the Mean Absolute Error (MAE) between all the PPG signal metrics extracted under the various motion artifact and the “still” stationary PPG signal baseline. As we can see from Fig. 9, for the majority of the PPG signal metrics, there are statistically significant differences between the still baseline and most of the artifacts. As expected, more intense head/face movements, like tilt and mouth stretch, yield greater differences in the signal metrics computed against the still baseline. This is much more evident while looking at full-body movements. Besides, a comparison of data from the left (??) and right (??) ear hints at differences between the PPG signals collected from the two ears. Multi-site PPG signals from the ears have been largely understudied so far. We believe our dataset is the perfect starting point to further explore this area.

## Usage Notes

### Data pre-processing

The data recorded from the Zephyr does not require additional processing as they are already pre-processed (with the exception of the ECGAmplitude and the BRAmplitude, which can be easily pre-processed using NeuroKit library).

However, the data collected from our earable prototype requires pre-processing. Firstly, the raw accelerometer data has to be converted to milli-g units by multiplying with 0.061, and the raw gyroscope data has to be converted to milli-dps (degrees per second) by multiplying with 17.5. This converts the raw IMU sensor data from an integer format to a more usable/standard format (i.e., milli-g and milli-dps). We then remove the direct current (DC) offset from the gyroscope data by applying a Butterworth band-pass filter (0.4–4 Hz cutoff). Secondly, the PPG signals can be pre-processed using bandpass filtering options available in Heartpy or NeuroKit libraries to extract HR, SpO_2_, etc.

### EarSet dataset

The EarSet dataset is available in^43^. Convenient libraries to pre-process and clean the physiological signals include HeartPy (https://python-heart-rate-analysis-toolkit.readthedocs.io/en/latest/) to extract heart rate data from PPG or ECG sensors, NeuroKit (https://neurokit2.readthedocs.io/en/latest/index.html) and BrainFlow (https://github.com/brainflow-dev/brainflow) to analyze PPG and ECG signals.

We believe that the EarSet dataset will foster research of new solutions to problems such as:

- *Motion Artifacts Filtering*: The dataset enables the exploration of how subtle head and face motions affect in-ear IMU and PPG signals. Firstly, this allows studying what kind of facial movements cause significant degradation of the PPG signals and how they might affect the accuracy of vital signs estimation. Secondly, the dataset will motivate the design of sophisticated filtering techniques for in-ear PPG signals - targeted at eliminating head and facial motion artifacts.
- *Sensor Location*: EarSet offers a unique opportunity to study whether the availability of PPG sensors in both ears could improve the estimation of vital signs. Having access to independent streams of PPG signals from the left and right ears could highlight asymmetries in the way people perform head and facial movements. These findings could be exploited to design improved signal-filtering approaches.
- *Sensor Configuration*: Given the need for low power consumption in future earable devices, the dataset allows the exploration of how different PPG hardware configurations (including 3 wavelengths), each with specific power requirements, affect the acquired PPG signal quality. This has important implications for the design of future devices and processing pipelines.
- *State-of-the-art Comparison*: The dataset contains several physiological measurements from ECG signals measured using a Zephyr Bioharness 3.0 chest strap. This enables validation and benchmarking of vital signs estimation methods applied to in-ear data with state-of-the-art methods from commercial devices unaffected by head/facial motions.

While the EarSet dataset opens up novel opportunities for earable devices, our approach still has a few limitations and presents opportunities for further improvements. Our focus is to offer a dataset to investigate the impact of head/face motions, in addition to full-body activities, on in-ear PPG signal quality and vital signs estimation. Skin tone is an additional factor that could affect data quality^35^. Although EarSet offers diversity in skin tones, the acquired data does not follow a uniform distribution among the six categories of pigmentation^29^. Future work will consider expanding the dataset to include additional participants to uniformly cover all skin tones.

All our participants were healthy at the time of the data collection and had no heart-related conditions. Future data collection efforts will consider participants with underlying conditions that could affect the morphology of the PPG signal even without the presence of motion-related artifacts. Correctly distinguishing the two cases would significantly increase the trustworthiness of earable devices beyond commercial settings - with the potential to be applied in clinical settings. Additionally, manual assessment of the PPG signal quality from experts in the field would complement the dataset, enabling the development of automatic pipelines to estimate expert-grade clinical assessments.

## Data Availability

We provide the raw data files obtained during the data collection structured by a user identifier. We did not implement any specialized code to pre-process the data.
